# Web-based alcohol intervention for Mäori university students: double-blind, multi-site randomized controlled trial

**DOI:** 10.1111/j.1360-0443.2012.04067.x

**Published:** 2012-11-07

**Authors:** Kypros Kypri, Jim McCambridge, Tina Vater, Steven J Bowe, John B Saunders, John A Cunningham, Nicholas J Horton

**Affiliations:** 1 Centre for Clinical Epidemiology and Biostatistics, School of Medicine and Public Health, University of NewcastleCallaghan, NSW, Australia; 2 Injury Prevention Research Unit, University of OtagoDunedin, New Zealand; 3 Faculty of Public Health & Policy, London School of Hygiene & Tropical MedicineLondon, UK; 4 Centre for Addiction and Mental HealthToronto, ON, Canada; 5 Centre for Behavioural Research in Cancer, Cancer Council VictoriaMelbourne, Vic., Australia; 6 Disciplines of Psychiatry and Addiction Medicine, University of SydneySydney, NSW, Australia; 7 Department of Mathematics and Statistics, Smith CollegeNorthampton, MA, USA

**Keywords:** Alcohol, brief intervention, feedback, indigenous health, internet, students

## Abstract

**Aims** Like many indigenous peoples, New Zealand Māori bear a heavy burden of alcohol-related harm relative to their non-indigenous compatriots, and disparities are greatest among young adults. We tested the effectiveness of web-based alcohol screening and brief intervention (e-SBI) for reducing hazardous drinking among Māori university students.

**Design** Parallel, double-blind, multi-site, randomized controlled trial.

**Setting** Seven of New Zealand's eight universities.

**Participants** In April 2010, we sent e-mail invitations to all 6697 17–24-year-old Māori students to complete a brief web questionnaire including the Alcohol Use Disorders Identification Test (AUDIT)-C, a screening tool for hazardous and harmful drinking. Those screening positive were computer randomized to: <10 minutes of web-based alcohol assessment and personalized feedback (intervention) or screening alone (control).

**Measurements** We conducted a fully automated 5-month follow-up assessment with observers and participants blinded to study hypotheses, design and intervention delivery. Pre-determined primary outcomes were: (i) frequency of drinking, (ii) amount consumed per typical drinking occasion, (iii) overall volume of alcohol consumed and (iv) academic problems.

**Findings** Of the participants, 1789 were hazardous or harmful drinkers (AUDIT-C ≥ 4) and were randomized: 850 to control, 939 to intervention. Follow-up assessments were completed by 682 controls (80%) and 733 intervention group members (78%). Relative to controls, participants receiving intervention drank less often [RR = 0.89; 95% confidence interval (CI): 0.82–0.97], less per drinking occasion (RR = 0.92; 95% CI: 0.84–1.00), less overall (RR = 0.78; 95% CI: 0.69–0.89) and had fewer academic problems (RR = 0.81; 95% CI: 0.69–0.95).

**Conclusions** Web-based screening and brief intervention reduced hazardous and harmful drinking among non-help-seeking Māori students in a large-scale pragmatic trial. The study has wider implications for behavioural intervention in the important but neglected area of indigenous health.

## Introduction

New Zealand was founded on the basis of a treaty between the indigenous (Māori) peoples and those, largely from Great Britain, who were part of the colonial expansion of European peoples 1. Today in New Zealand, Māori have significantly poorer health, with a life expectancy 8 years less than non-Māori 2. One contributor to this inequality is hazardous consumption of alcohol 3. Māori have more than twice the prevalence of episodic heavy drinking 2 and an alcohol-attributable death rate more than four times that of non-Māori 2. These disparities are greatest among those aged less than 30 years 3. New Zealand alcohol policy was liberalized dramatically in the 1980s and 1990s, resulting in increased physical availability of alcohol, a reduction in the minimum purchase age and longer trading hours 4. In the face of weak supply-side policies, interventions to reduce demand for alcohol that can be widely disseminated are of crucial importance to reduce alcohol-related harm.

Evidence from systematic reviews and meta-analyses suggests modest effect sizes of computerized interventions delivered to university students; however, many of the trials were methodologically flawed and interventions were delivered in conditions that could not be integrated within routine health-care or health promotion practice 5–8. There have been no large-scale trials of such interventions among indigenous people.

Previous trials in New Zealand 9,10 have shown that web-based alcohol screening and brief intervention (e-SBI) delivered in the primary health-care setting can be effective in reducing hazardous drinking among university students, a particularly high-risk group 11. Estimating the effectiveness of e-SBI separately for Māori was not possible in those trials, because participants were recruited from among those presenting to the student health service where Māori comprised only 7% of patients. In the present study we adopted a proactive recruitment approach based on THRIVE (Tertiary Health Research Intervention via E-mail), an e-SBI programme developed in Australia 12,13, and survey methods developed in New Zealand 14 in which thousands of students are invited to participate from university enrolment databases. We used this approach to ensure equal explanatory power for Māori, who have traditionally been served poorly by health research 15, and to create a sustainable platform for health promotion activities at universities. The aim was to estimate the effectiveness of e-SBI in reducing hazardous and harmful drinking among Māori university students. We hypothesized that, relative to screening alone, e-SBI would reduce alcohol consumption and related problems.

## Methods

Ethical approval for the study was given by New Zealand's Multi-region Ethics Committee (MEC/10/01/009).

### Trial design

**Figure 1:**
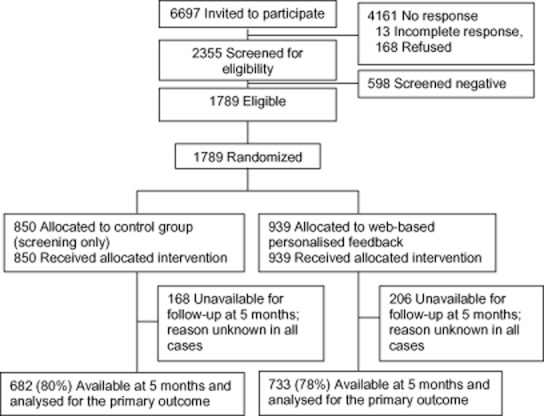
Trial flow-chart. Analyses incorporated participants with a post-randomization response. In addition, a sensitivity analysis utilizing multiple imputation incorporated all randomized participants in the analysis

The e-SBINZ study involves parallel randomized controlled trials: one involving Māori and the other non-Māori university students 16. The Māori trial reported here was a multi-site, double-blind, parallel groups randomized controlled trial with a 1 : 1 allocation ratio (Fig. 1).

### Participants

Participants were university students who indicated that they were Māori in response to the ethnicity question on the university enrolment form and who were aged 17–24 years at the time they were invited (some of whom turned 25 between then and when they participated). All data were collected via the internet, such that participants could answer screening questions, participate in the intervention and complete follow-up assessments wherever they chose. We invited all eight New Zealand universities to participate, but one did not because of internal rules that did not permit the research team to invite students directly by e-mail, as per the study protocol.

### Sample size

We based the estimate of required sample size on the 6-month outcomes in the THRIVE trial 17. Assuming a 5% level of significance, 80% power, a dispersion factor of 0.92 and attrition of 30%, 547 participants per group were required at follow-up 16. Assuming that 40% would agree to be screened, a conservative estimate in light of the THRIVE trial 17, we sought to invite 7814 Māori students aged 17–24 years. The university enrolment data showed that there were only 6697 Māori students in this age group enrolled across the seven universities. As shall be shown below, eligibility rates, participation and retention in the trial turned out to be sufficiently high to more than offset this deficiency.

### Recruitment and screening

On 19 April 2010, all 6697 students were invited to participate using recruitment procedures described in detail elsewhere 14. In summary, up to three reminder e-mails were sent during the following weeks. Students were offered the opportunity to win a NZ$500 supermarket voucher or an Apple iPad by participating. Respondents visited a website consisting of a branched three-page questionnaire with items covering: (i) gender, age and living arrangements; (ii) drinking in the last 12 months (yes/no); and (iii) the Alcohol Use Disorders Identification Test (AUDIT)-C, a validated three-item screening tool for hazardous and harmful drinking 18. We limited the screening to three questions because there is review-level evidence that asking questions about alcohol consumption can itself influence behaviour, producing reductions in self-reported drinking levels 19. This evidence base is stronger among university students than in other populations, and suggests the possibility of reactivity to the research conditions and possible bias towards the null 19.

### Randomization and blinding

Students were sent an e-mail containing a hyperlink to a web questionnaire and were informed that: ‘the main focus of this study is student alcohol use over time and its consequences’. Respondents who scored ≥4 were assigned via simple randomization by the web server to the control group (screening only) or intervention. This procedure was to ensure that participants were blind to the true nature of the study, which was presented as two surveys, in order to minimize the potential for performance bias 20. Researchers were blind to participants' group allocation, as randomization and all other study procedures were fully automated and thus could not be subverted.

### Intervention

The AUDIT-C comprises the three consumption questions of the 10-item World Health Organization (WHO) AUDIT 21. Those in the intervention group were then asked AUDIT items 4–10, all of which are concerned with alcohol problems, and additional questions on the largest number of standard drinks consumed on one occasion in the last 4 weeks, the duration of the drinking episode in hours, and their body weight, for the purpose of estimating peak blood alcohol concentration (BAC). They then completed the 10-item Leeds Dependence Questionnaire (LDQ) 22. All these questions were presented as a seamless series of web pages immediately after screening and randomization. The psychometric performance online of both the AUDIT and the LDQ has been confirmed in a previous study with university students 23.

The intervention group received personalized feedback consisting of: their AUDIT and LDQ scores with an explanation of the associated health risk and information about how to reduce that risk; an estimated BAC for their heaviest episode in the previous 4 weeks, with information on the behavioural and physiological sequelae of various BACs, and traffic crash relative risk; estimates of monetary expenditure per month; bar graphs comparing the reported episodic and weekly consumption levels with those of other students and the general population of the same age and gender; and hyperlinks for help with drinking problems. Further web pages were presented as options, offering facts about alcohol, tips for reducing the risk of alcohol-related harm, and where medical help and counselling support could be found. A demonstration version of the instrument can be viewed at http://www.webcitation.org/69vNZW3BA.

The intervention was developed iteratively over a 10-year period involving consultation with Māori and non-Māori university students, Māori student support services and with the aid of Māori co-investigators and research staff. This consultation and research yielded an instrument that was appealing to Māori and non-Māori university students such that content was not specific to either group. Notably, on the basis of advice from Māori co-investigators, Māori-specific normative feedback was eschewed to avoid framing Māori student drinking in terms of a deficit model 24.

### Outcomes and follow-up

Five months after randomization, in September 2010, all participants were sent a pre-notice letter and then an e-mail 2 days later containing a hyperlink to a web-based follow-up questionnaire. Questions concerned the frequency of drinking and amount consumed per typical drinking occasion, all with a reference period of the last 4 weeks. These frequency/quantity measures have been validated extensively 25 and used with this population group 14. In addition, participants were presented with the five-item Academic Role Expectations and Alcohol Scale (AREAS) 26, an alcohol problems measure also validated online with university students 23. The AREAS asks: ‘As a result of drinking alcohol, how often have you experienced each of the following over the past 4 weeks: you were late for a class, you missed a class, you were unable to concentrate in class, you failed to complete an assignment on time?’ with response options: once, twice, three times, four or more times; and then ‘How much do you think your drinking negatively impacted how much you learned, or your grades?’ with response options: not at all, a little, quite a lot, a great deal.

There were four planned primary outcome measures: frequency of drinking (range: 0–28 days), number of standard drinks (10 g ethanol) per typical occasion, average weekly volume [(28-day frequency × typical quantity)/4], and AREAS score (range 0–15). Secondary outcomes included the prevalence of drinking above New Zealand recommended limits for acute risk (for women and men, respectively, more than four and more than six standard drinks on one occasion in the preceding 4 weeks) and chronic risk (for women and men, respectively, more than 14 and more than 21 standard drinks per week in the preceding 4 weeks) 27.

### Statistical analysis

The analysis plan was constructed a priori, and is described in the trial protocol 16. The four primary outcomes (frequency, quantity, volume and AREAS scores) were analysed with negative binomial regression with empirical variance using STATA version 10.1. For analyses of the two secondary outcomes (proportions of students exceeding drinking guidelines) we used logistic regression models. The results are presented as risk ratios and odds ratios, respectively.

Participants were analysed in the groups to which they were randomized (intention to treat, ITT). We describe patterns of missing values as well as comparing those observed and those missing in terms of baseline characteristics. We compared the baseline AUDIT-C scores, age and gender of participants lost to follow-up versus those followed-up to assess whether loss to follow-up was differential by randomization group.

We fitted two types of models for the ITT analysis. The first yields unbiased effect estimates under the assumption that values are missing at random (MAR) 28. In the second, we used the rctmiss command in STATA to conduct a missing not at random (MNAR) sensitivity analysis with the outcome variable in which the largest effect was observed (volume). We fitted a sensitivity analysis with a parameter *δ* that allowed for a difference between unobserved and observed in the group with the larger fraction of missing information. This model allowed there to be a difference between observed and unobserved participants in the intervention group and assumes that observed and unobserved participants in the control group are identical, i.e. conditions that would produce attrition bias. The value *δ* is the multiplicative factor which controls this MNAR mechanism: the unobserved drink exp(*δ*) × that of those observed in the intervention group (when *δ* = 0 this is equivalent to a missing at random assumption).

To account for having four primary outcomes we set the significance level to α = 0.05/4 = 0.0125. For the purpose of comparison with several recent systematic reviews and meta-analyses, we computed a Cohen's *d* as a measure of the effect size for each of the primary outcomes 1988.

We conducted four *post-hoc* subgroup analyses examining whether gender, age, AUDIT-C score and university modified the effect of the intervention on the four primary outcomes. Each of these variables was included in the regression models using the testparm command in STATA, which produces a χ^2^ statistic for non-linear models.

## Results

### Screening and randomization

Participant flow, follow-up rates and the numbers analysed are presented in Fig. 1. Of 6697 e-mail invitations, 2355 (35%) completed screening (Table 1). Of these, 1789 (75%) screened positive for hazardous or harmful drinking and were randomized to control (*n* = 850) or intervention (*n* = 939). The median completion time for the baseline questionnaire was 1.2 minutes [interquartile range (IQR) 0.9–1.7] and the intervention took a further 4.3 minutes (IQR 3.3–5.5) plus reading time, which was not measurable but is expected to have been less than 5 minutes. Table 2 presents summary data illustrating the equivalence of the two study groups at baseline despite the surprisingly large difference in the numbers allocated randomly to each group, which has a 2% probability of occurring by chance in a binomial distribution. Careful checking of the computer program confirmed that randomization was implemented correctly.

**Table 1:** Screening participation rates, age and drinking data by university.

University	Number of eligible students^a^	Number (%) screened	Number (%) women	Mean age (SD)	Mean AUDIT-C score (SD)
A	994	417 (42.0)	280 (67.2)	19.9 (1.7)	6.8 (2.5)
B	1408	619 (44.0)	391 (63.2)	20.2 (1.8)	5.2 (2.7)
C	603	214 (35.5)	133 (62.2)	20.1 (1.8)	5.9 (2.6)
D	90	34 (37.8)	22 (64.7)	19.3 (1.4)	6.6 (2.8)
E	1116	316 (27.1)	234 (74.1)	20.4 (2.0)	5.7 (2.8)
F	1180	269 (22.8)	186 (69.1)	20.3 (1.7)	5.7 (2.6)
G	1256	486 (38.7)	336 (69.1)	20.2 (1.8)	6.1 (2.8)
Total	6697^b^	2355 (35.2)	1582 (67.2)	20.2 (1.8)	5.9 (2.7)

a Students of Maori ethnicity aged 17–24 years at the time of invitation.

b Women comprised 60% of the Maori university student population aged up to 24 years in 2010 (http://www.educationcounts.govt.nz/statistics/tertiary_education/participation, accessed 15 August 2012). AUDIT: Alcohol Use Disorders Identification Test; SD: standard deviation.

**Table 2:** Baseline demographic and drinking characteristics of trial participants

	Control (n *=* 850)	Intervention (n *=* 939)
Females	66.8%	64.3%
Mean age (SD)	20.1 (1.7)	20.2 (1.8)
Mean AUDIT-C score (SD)	6.9 (2.0)	6.9 (2.0)
Drinking summary data^a^		
Drinks alcohol two or more times per week	28.0%	30.6%
Mean standard drinks per typical drinking occasion (SD)	8.4 (4.6)	8.4 (5.3)
Drinks six or more drinks per occasion weekly or more often	38.4%	39.0%

From Alcohol Use Disorders Identification Test (AUDIT)-C items. SD: standard deviation.

### Follow-up assessment

At follow-up, data were obtained from 682 participants in the control group (80%) and 733 in the intervention group (78%). These included 18 control group participants and 28 intervention group participants who provided follow-up data by e-mail rather than via the website. The median time from sending e-mail invitations to completion of follow-up was 2 days (IQR: 1–8 days) in each group.

Loss to follow-up was not differential by group and measured covariates were equivalent across the groups: among those unobserved at follow-up, women comprised 65 and 63% of the control and intervention groups, respectively (*P* = 0.59). The mean age of those unobserved was 19.8 and 20.1 years, respectively (*P* = 0.11), and mean AUDIT-C scores were 7.3 and 7.1, respectively (*P* = 0.35).

Mean baseline AUDIT-C scores were slightly higher among those unobserved at the follow-up [mean difference 0.37 points, 95% confidence interval (CI): 0.14–0.60]. Unobserved participants were significantly younger than those observed (mean difference −0.24 years, 95% CI: −0.42 to −0.04 years).

Table 3 presents all outcome data. It can be seen that the majority of this population exceeds thresholds for acute harm (binge drinking) but they drink infrequently (just over once a week on average), thus less than one in five exceed guidelines for chronic harm.

**Table 3:** All outcome data.

Outcome (total n *=* 1415)	Median (25th and 75th percentiles)			
Control		Intervention	
n *=* 682		n *=* 733	
Frequency of drinking (no. of days drinking in the last 4 weeks)	5	(3–8)	4	(2–7)
Typical occasion quantity (no. of drinks per typical drinking occasion)	6	(3–9)	5	(3–8)
Volume consumed (no. of drinks per week)	6	(3–13)	5	(2–12)
Academic-related alcohol problems (AREAS) score	1	(0–2)	1	(0–2)
Exceeded guidelines for avoiding acute harm^a^	55.6%		51.5%	
Exceeded guidelines for avoiding chronic harm^b^	19.5%		14.8%	

All measures use the preceding 4 weeks as the reference period.

a Alcohol Advisory Council (New Zealand): no more than four drinks (40 g ethanol) in any one occasion for women, and no more than six drinks (60 g ethanol) in any one occasion for men.

b No more than 14 drinks (140 g ethanol) per week for women, and no more than 21 drinks (210 g ethanol) per week for men. AREAS: Academic Role Expectations and Alcohol Scale.

#### Primary outcomes

Table 4 presents results for all primary and secondary outcomes. There were statistically significant effects in the main analysis for three of the four primary outcomes which were robust to the Bonferroni correction for multiple statistical tests. Twelve participants (seven control, five intervention) reported extreme values (>30) for the number of drinks consumed per typical occasion. With these cases removed, the effect estimate for this outcome attenuated from −8% to −7% (*P* = 0.039). Cohen's *d* was 0.13 (95% CI: 0.03–0.24), 0.09 (95% CI: 0.01–0.20), 0.16 (95% CI: 0.06–0.27) and 0.13 (95% CI: 0.02–0.23), respectively, for the primary outcomes in the main analysis.

**Table 4:** Intervention effects.

	Intervention/control	
Primary outcomes^a^		
Frequency of drinking (*n* = 1415)	RR = **0.89**	(0.82–0.97)
		*P* = 0.01
Typical occasion quantity (*n* = 1414)	RR = 0.92	(0.84–1.00)
		*P* = 0.04
Volume of alcohol consumed (*n* = 1414)	RR = **0.78**	(0.69–0.89)
		*P* < 0.001
Academic problems (*n* = 1368)	RR = **0.81**	(0.69–0.95)
		*P* = 0.01
Secondary outcomes^b^		
Odds of binge drinking: risk of acute harm (*n* = 1414)	OR = 0.80	(0.64–1.01)
*P* = 0.06
Odds of heavy drinking: risk of chronic harm (*n* = 1414)	OR = 0.65	(0.48–0.88)
*P* < 0.001

RR: rate ratio; 95% CI: confidence interval.

a Relative risk ratios adjusted for baseline Alcohol Use Disorders Identification Test (AUDIT)-C score with 95% confidence intervals, from negative binomial regression models.

b Odds ratios (OR) adjusted for baseline AUDIT-C score with 95% confidence intervals, from logistic regression models. Significant results of the primary analysis after the Bonferroni adjustment for the four primary outcomes (where *P* < 0.0125) are shown in bold type.

#### Sensitivity analysis

For a value of delta equal to 0.3, the model yielded results with *P*-values similar to the adjusted alpha level (*Z* = 0.158699/0.064768 = 2.45, *P* = 0.0143). This corresponds to a model where unobserved intervention subjects were drinking 1.35 times [exp(0.3)] as many drinks as observed intervention subjects, while unobserved controls were the same as observed controls.

#### Secondary outcomes

Relative to controls, the intervention group had a statistically significant lower prevalence of exceeding recommended limits for chronic harm but not acute harm. They were 35% less likely to exceed recommended weekly consumption limits.

The subgroup analyses revealed no significant variation in the effects of the intervention by age, gender or drinking level on the primary outcomes. There was a difference in the intervention effect by university on the AREAS outcome (χ^2^ = 13.01, d.f. = 6, *P* = 0.043); however, considering the multiple tests performed, this result is not statistically significant.

## Discussion

Hazardous or harmful drinkers who received e-SBI drank 22% less alcohol than controls 5 months after randomization, and their alcohol problem scores were 19% lower. Both these effects were maintained under conservative analytical assumptions. The differences in overall volume consumed were driven principally by reductions in the frequency of drinking, although there was also some evidence of reduced quantity per drinking occasion and there was a large reduction in the prevalence of drinking above guidelines for chronic harm.

The rate of attrition (21%) was lower than that seen in the THRIVE trial, on which this trial was based, where 35% of undergraduates receiving e-SBI were lost to follow-up 6 months after intervention 17, and indeed is lower than in any entirely online behavioural intervention trial of which we are aware. Those lost to follow-up were similar with regard to gender but were slightly younger and heavier drinkers. In no analysis was there differential attrition by randomized group. The sensitivity analysis showed that the results are fairly robust to assumptions about missingness, as it would be improbable that those lost to follow-up are sufficiently different in the intervention versus control groups to account for the observed effects.

In a previous study using a similar e-SBI instrument we found an assessment effect, i.e. hazardous or harmful drinkers who received 10 minutes of web-based assessment of their drinking, in the absence of a feedback intervention, subsequently reported drinking less than a screening-only control group 30. On the basis of those results, and systematic review findings 19, we sought to minimize assessment of the control group in the present trial by requesting only demographic information and asking the three questions of the AUDIT-C, which took, on average, 1.2 minutes to complete. It has been suggested that by focusing attention on their drinking, assessment may encourage participants to monitor and then modify their behaviour 30. Such assessment effects have been found in relation to the 10 items of the AUDIT alone 31 so it remains possible, although difficult to evaluate how likely, that the intervention effect has been underestimated in this trial.

Given the relatively small size of the Māori student population, we had to invite all eligible individuals for screening. Accordingly, contamination may have occurred if those who received the intervention discussed their feedback with fellow students or influenced behaviour in some other way in the control group. This, too, would have biased effect estimates towards the null (Type II error).

Laboratory measures of alcohol-related harm were not appropriate, because biomarkers are insensitive to the episodic heavy drinking characteristic of young people 32. It was also judged potentially counterproductive to seek consent from participants to obtain access to health service records, given that contact was so brief and entirely web-based and the population were not seeking treatment. Accordingly, we relied on self-report, which is considered sufficiently reliable for alcohol treatment trials 32. In addition, there is evidence of greater candour in the reporting of stigmatized behaviours, including hazardous and harmful drinking, when elicited via computers compared with pen-and-paper methods 33. It does, however, remain possible that participants receiving the intervention were inclined to under-report their drinking to a greater extent than controls, which would have biased estimates away from the null. Such a possibility cannot be ruled out with this design and is challenging to evaluate rigorously.

Overall, the observed effects were similar to those found using a similar intervention with university students (7% of whom were Māori) presenting to a health service in New Zealand 10. One important effect size in this study—a 22% difference in weekly drinking at follow-up—is larger than that reported in a systematic review of conventionally delivered face-to-face brief interventions in primary health care (13%) 34. The range of primary outcome effect sizes measured as Cohen's *d* (0.10–0.17) is very similar to the range of estimates (0.09–0.16) obtained in a meta-analysis of a variety of computerized interventions for drinking among university students, conducted primarily in laboratory conditions or in settings unlikely to be scaleable to widespread implementation 5–8.

No previous research has examined the efficacy or effectiveness of alcohol screening and brief intervention in an indigenous population trial. Our findings have implications for the provision of health promotion services and the conduct of related research with indigenous peoples in the United States, Canada and Australia, many of whom suffer similarly elevated alcohol-related mortality 35,36. This population group is relatively well educated such that findings may not generalize to other indigenous population groups; however, the findings show that it is possible to proactively reach a large number of indigenous drinkers via the internet and engage them in reflection upon their drinking, leading to reductions of public health significance.

Screening participation rates are rarely reported in randomized controlled trials, such that it is impossible to know to whom effect estimates generalize. The design of this national trial, in which screening rates could be recorded, permits the quantification of generalizability to the third or so of Maori students willing to complete a survey upon a simple e-mail invitation. There is evidence from large-scale web-based research projects in the New Zealand university setting that participation of more than 80% can be achieved with pre-notice letters and telephone reminders 9 and approximately 65% with pre-notice letters alone 37, but such resources are not affordable for annual screening programmes.

An orientation in the design of the intervention towards sustainable implementation makes the likelihood of attaining public health benefit a clear strength of the study. The e-mail invitation is practically free (the cost of e-mail traffic only) and could be issued each semester by universities. e-SBINZ was delivered via open source software (http://www.limesurvey.org/) that can be easily modified and, as in this trial, housed on a single server for an entire country. An aspiration underlying this programme of research has been to bridge the evidence–practice gap, and in this regard it is worth noting that upon receipt of these findings all New Zealand's universities resolved to implement e-SBINZ routinely from 2012.

### Clinical trial registration

ACTRN12610000279022.

### Declarations of interest

None.
